# Foreign Bodies in the Ear

**Published:** 1910-04-02

**Authors:** 


### FOREIGN BODIES IN THE EAR.

Foreign bodies are found most frequently in the ears of children, who put them in intentionally; their presence in adults is generally accidental, except that a pledget of cotton-wool may find its way deeply into the canal and be forgotten, and plugs of tobacco or some other substance are sometimes put into the ear by the ignorant in the hope of relieving earache.

The symptoms caused by a foreign body vary very greatly; many do little or no harm, and unfortunately more damage is sometimes caused by injudicious attempts at removal than by the presence of the body. It is important to remember that there is no need for hurry in the removal of an inert body unless it is causing pain by pressing on the drum, or blocking discharge from a suppurating tympanum. The symptoms depend chiefly on the nature of the substance: (1) Inert bodies which do not absorb moisture nor irritate; (2) those which absorb moisture and so become swollen or decompose; (3) bodies which cause irritation chemically or by having sharp edges; and (4) living bodies, such as fleas, flies, or maggots. A smooth inert body which does not completely block the meatus may remain for an indefinite time without producing symptoms; if the lumen be obstructed there will be some deafness and tinnitus, if the substance swell and cause pressure on the meatus there will be pain, and if it press upon the drum the pain is usually very severe. A small insect moving on the drum causes intense agony. A foreign body, especially if it absorb moisture and decompose, may set up marked inflammation and swelling of the walls of the meatus. If it ruptures the drum an acute otitis results and, if the outflow of discharge be obstructed, serious complications are likely to follow; the same danger is present if there is a previous suppurative otitis. Foreign bodies occasionally, though rarely, lodge in the tympanum, being either forced through the drum or passing through an old perforation. A variety of live insects may find their way into the external meatus; small flies, gnats, and fleas are the most common, and several cases have been recorded in which cockroaches have crawled into the ear while the patient was sleeping. The larvæ of flies are hardly ever found except in cases of very dirty and neglected aural suppuration, for the smell of decomposition attracts the insect to lay its eggs in the ear; they are fortunately rare in this country. Insects usually cause much discomfort by their movements on the drum, and may produce great pain by stinging. The presence of maggots is generally associated with much external otitis and swelling of the canal, as well as with severe otitis media, and the pain is often very severe.

In connection with foreign bodies, some points in the anatomy of the external auditory meatus must be mentioned. The canal is about an inch long, of which a little less than half is cartilaginous, the inner part being bony. The bony portion is relatively shorter in childhood and is represented at birth only by the tympanic ring. The tube is narrowest near its middle, at the junction of the bony and cartilaginous parts. This is called the "isthmus," and is the locality where a foreign body is especially likely to be impacted; but if it pass beyond this point the difficulty of extraction is greatly increased. The meatus is directed forwards and inwards, and is curved on vertical section. The isthmus is thus near the highest part of the canal, which makes a very acute angle with the lower and front part of the drum, and so forms a kind of pocket, the "sinus" of the meatus where a small foreign body is liable to lodge; in this position it is not only difficult to remove, but it may easily escape detection.

The removal of foreign bodies is not a matter of great difficulty in the vast majority of cases, but in the few exceptions it requires great perseverance and considerable ingenuity. Two points must be remembered as essential, to use a good light and so make sure of the presence and position of the body, and to do no harm by attempts at extraction. Unless there is retention of pus there is no need for haste and, if the meatus is much swollen, it is sound practice to apply a leech in front of the tragus and dry heat by means of a roll of flannel, and postpone attempting the removal until next day. Syringing should always be tried first, and perseveringly before using instruments unless (1) the syringing causes giddiness, (2) the body is pointed and likely to injure the drum, or (3) there is a large perforation and a small foreign body which may be washed through into the tympanum. A fine nozzle should be used and the stream directed to any gap between the meatal walls; if it fails the patient should lie with the meatus directed downwards, so that gravity may assist in the removal. If still unsuccessful it is wise to wait and repeat on the following day. If it still fails instrumental extraction is necessary with various forms of hooks and forceps, and for this a good light and great gentleness are essential. In children, at any rate, an anæsthetic is necessary for this, and often also for effective syringing. Sometimes a smooth round body, such as a bead or a pebble, may be removed by applying a small brush dipped in glue and packing it in position until set. Insects may be floated out by filling the ear with warm oil, or they may be syringed out after first killing them by dropping in rectified spirit or biniodide solution, or after stupefying them with a pledget of wool moistened with chloroform. Larvæ often remain firmly attached, and must be removed with forceps. Occasionally, when a foreign body is very firmly impacted, or lies out of reach in the sinus of the meatus, or is so sharp as to cause great risk of perforation of the drum, it is necessary to turn the auricle forward and separate the membranous from the osseous meatus, as in the radical mastoid operation, after which the extraction can be performed without difficulty.

